# Insulin-Like Growth Factors Promote Vasculogenesis in Embryonic Stem Cells

**DOI:** 10.1371/journal.pone.0032191

**Published:** 2012-02-21

**Authors:** Stephanie M. Piecewicz, Ambarish Pandey, Bhaskar Roy, Soh Hua Xiang, Bruce R. Zetter, Shiladitya Sengupta

**Affiliations:** 1 Department of Medicine, Brigham and Women's Hospital, Harvard Medical School, Boston, Massachusetts, United States of America; 2 Harvard-MIT Division of Health Sciences and Technology, Cambridge, Massachusetts, United States of America; 3 Department of Medical Science and Technology, Indian Institute of Technology, Kharagpur, India; 4 Vascular Biology Program and Department of Surgery, Children's Hospital, Harvard Medical School, Boston, Massachusetts, United States of America; University of Padova, Medical School, Italy

## Abstract

The ability of embryonic stem cells to differentiate into endothelium and form functional blood vessels has been well established and can potentially be harnessed for therapeutic angiogenesis. However, after almost two decades of investigation in this field, limited knowledge exists for directing endothelial differentiation. A better understanding of the cellular mechanisms regulating vasculogenesis is required for the development of embryonic stem cell-based models and therapies. In this study, we elucidated the mechanistic role of insulin-like growth factors (IGF1 and 2) and IGF receptors (IGFR1 and 2) in endothelial differentiation using an embryonic stem cell embryoid body model. Both IGF1 or IGF2 predisposed embryonic stem to differentiate towards a mesodermal lineage, the endothelial precursor germ layer, as well as increased the generation of significantly more endothelial cells at later stages. Inhibition of IGFR1 signaling using neutralizing antibody or a pharmacological inhibitor, picropodophyllin, significantly reduced IGF-induced mesoderm and endothelial precursor cell formation. We confirmed that IGF-IGFR1 signaling stabilizes HIF1α and leads to up-regulation of VEGF during vasculogenesis in embryoid bodies. Understanding the mechanisms that are critical for vasculogenesis in various models will bring us one step closer to enabling cell based therapies for neovascularization.

## Introduction

Stem cell differentiation into endothelial cells is the first step of vasculogenesis. [1]–[2] This process occurs spontaneously *in vitro* in embryonic stem cell derived embryoid bodies (EB). [3] The formation of vascular channels in EB closely mimics vasculogenesis *in vivo*. [4] Many groups have attempted to preferentially drive endothelial differentiation from embryonic stem cells but the process remains inefficient or labor intensive. [5]–[6] Several critical signaling factors have been identified, including , TGF-β, BMP4, and VEGF, that drive the differentiation of pluripotent stem cells first into mesoderm, endothelial progenitor cells and finally into mature endothelium. [7]–[8] In a recent study, we demonstrated that the cell surface glycome plays a critical role in this process. [9] However, a better understanding of the signaling pathways that control vasculogenesis is needed for efficient endothelial differentiation.

Insulin-like growth factor (IGF) signaling mediates many critical cell responses including mitogenesis, proliferation, growth, differentiation, and angiogenesis. [10]–[12] IGFs circulate in the bloodstream at nanomolar concentrations and are generally bound to one of six IGF binding proteins (IGFBPs), which regulate their availability to cell surface receptors. [13] Glycosaminoglycans also play a critical role in liberating the ligands from their binding proteins and making them available to bind receptors. [14] Insulin-like growth factors 1 and 2 (IGF-1 and IGF-2) share about 50% of amino acid sequence with insulin and have some affinity for the insulin receptor, but have distinct physiological functions from insulin mediated by signaling through the IGF-1 receptor. [15] Binding to this tyrosine kinase receptor activates downstream PI3K and MAPK cascades that stimulate growth and survival of particular cell types. IGF1R binds IGF1, IGF2, and insulin with decreasing affinity. The IGF-2 receptor (Man-6-P) is not a tyrosine kinase, and whether it elicits unique downstream signaling pathways or acts as a ‘sink’ for IGF-2 is relatively unknown. [16]–[18]

Insulin like growth factors are essential for embryonic development. Both IGF1 and IGF2 are necessary to maintain normal embryonic growth rates in mice and do not compensate completely for one another. [19] Indeed, IGF2 has been shown to play specific roles in adult development and disease. [20] IGF1 mutant mice display developmental bone defects and reach only 70% the size of wild-type mice. In contrast, overexpression of IGF2 leads to fetal overgrowth and phenotypes similar to Beckwith-Wiedemann Syndrome. [21] Complete absence of IGF1R or of both IGF1 and IGF2 is embryonic lethal. While several studies have shown a connection between IGFs and neovascularization, their specific role in vasculogenesis is largely unknown. IGF1 has been shown to induce neovascularization and endothelial proliferation in cornea and retina models. [22] Similarly, IGF1R signaling from IGFs has been shown to mediate neovascularization in human lung development and in zebrafish cardiovascular development. [23]–[24] Previous studies have implicated IGFs in stimulating endothelial progenitor cell differentiation, migratory capacity, homing and incorporation into existing vascular networks. [25]–[26] Mesoderm, the precursor of blood vessels, muscle, bone and other specific lineages, have also been shown to be dependent on insulin-like growth factors. [27] Interestingly, studies have also established a role for the insulin-like growth factor pathway in maintaining stem cell pluripotency. [28]–[29] In this study, we investigated the role of insulin-like growth factors 1 and 2 in embryonic stem cell vasculogenesis by focusing on endothelial differentiation in embryoid bodies. Our results establish that both IGF1 and IGF2 promote embryonic stem cell differentiation into endothelial cells acting through the IGFR1 pathway.

## Methods

### Cell Culture and Materials

Mouse embryonic stem cell line 9TR#1 strain 129 was obtained from ATCC. Embryoid bodies were induced by seeding cells at a density of 3100 cells/cm^2^ on neutrally charged tissue culture plates (Corning). Mouse recombinant IGF-1 and IGF-2 (R&D) were reconstituted in PBS and diluted to desired concentrations immediately before use. Picropodophyllin (Enzo Life Sciences) was reconstituted in DMSO and diluted to desired concentration immediately before use. For all experiments, IGF and PPP treatment doses were reconstituted to desired dose in differentiation media and added to cells for 3 hours, after which media was replaced with fresh differentiation media. Cells were treated daily with IGF-1 and IGF-2 beginning at day 1, and treated on alternate days with PPP, or as indicated in the text. Neutralizing antibodies for IGFR-1 and IGFR-2 (R&D) were added to EB (2 ng/mL) and incubated at 37°C for one hour before addition of 5 ng/mL IGF. Where indicated, cells were serum starved and treated every other day with 50 nM Rapamycin (Tocris) for 30 minutes prior to IGF treatment.

### Quantitative PCR

RNA was isolated at the indicated days using Aurum Total RNA isolation kit and total RNA reverse transcribed using iScript cDNA Synthesis kit. (Bio-Rad, Hercules, CA) The levels of mRNA for specific genes were detected by using designed oligonucleotide primers (IDT, Inc.) and monitoring SYBR Green fluorescence. The normalized reporter (Rn) was calculated from threshold values, C_t_, for each gene as follows: Rn = 2 X^−δ C^_T_, where δ C_T_ = (average C_Ttarget_-average C_Texperimental control_) and 18S was used as endogenous control to normalize quantities of cDNA.

### Immunohistochemistry

EB were transferred to 0.1% gelatin coated coverslips on day 3 and differentiated as indicated above and with various treatments as indicated. On day 7 (TIE-2) and day 10 (CD31), cells were fixed with 4% PFA. TIE-2 (Santa Cruz), CD31 (Abcam), Alexafluor®546 goat anti-rat (Invitrogen) Alexafluor®488 goat anti-rabbit (Invitrogen) were incubated with cells for 1 hour at room temperature (TIE-2) or overnight (CD31). Cells were counterstained with DAPI (Sigma). Images were obtained using a Nikon Eclipse TI microscope and NIS-Elements 3.2 software.

### Western Blotting

Indicated doses of PPP were added to cells and incubated for 4 hours, at which time cells were stimulated with 300 ng/mL IGF1. For the analysis of HIF1α protein levels, cells were serum-deprived overnight, and stimulated with indicated concentrations of IGF1 for 2 hours, with or without 30 minute Rapamycin treatment (50 nM). Primary antibodies Phosphorylated-IGFR1 Y1136, IGFR1, Phosphorylated AKT S473, AKT, P44/42 MAPK, actin (Cell Signaling), and ERK1/ERK2, HIF1α (Santa Cruz) were incubated with membranes overnight at 4°C. Quantification of protein levels was done using GeneTools ® software. Background was subtracted from protein raw values and then normalized to levels of actin.

### Flow Cytometry

Embryoid bodies were washed with PBS and disassociated using 0.25% Trypsin. Single cell suspensions were fixed in ice-cold methanol and incubated with rat monoclonal antibody against CD16/CD32 (Pharmigen, BD Biosciences) in order to block Fc receptors. Cells were incubated overnight with VWF primary antibody (DAKO) or isotype matched polyclonal IgG antibody (Santa Cruz). Cells were washed and incubated with Alexafluor488-conjugated secondary antibody and analyzed on a Accuri C6 Flow Cytometer using CFlow Plus Software.

### Statistical Analysis

All experiments were done in triplicate, unless otherwise noted, and repeated to ensure consistent results. Statistical analyses were performed by Prism ® software (GraphPad Software, San Diego, California) using one-way ANOVA and Newman-Keuls post-test correction with acceptance level p<0.05. Data is presented as mean +/− SEM.

## Results

### IGF1 and IGF2 Treatment Increases Mesoderm Development

Embryonic stem cells permitted to aggregate into cystic embryoid bodies (EB) spontaneously differentiate into the three germ layers which go on to differentiate into diverse lineages. Vascular channels form in EB after approximately one week and have been shown to recapitulate *in vivo* vascular development steps and are thus a robust model for studying vasculogenesis. [4]

**Figure 1:**
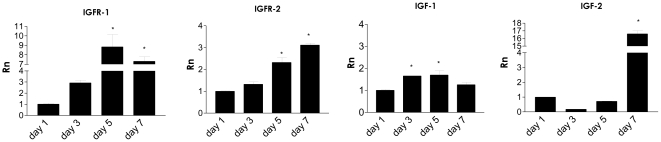
Expression of Insulin Like Growth Factors, Receptors, and Binding Proteins with Embryonic Stem Cell Differentiation. A) mRNA levels of IGFR-1, IGFR-2, IGF1, and IGF2 increase with time in differentiating embryoid bodies measured from days 1–7, although IGF1 appears to remain steady, pointing to its role in both pluripotency and differentiation. * denotes P<0.05 to day 1 control, Rn denotes normalized reporter.

As a first step, we examined the expression of IGF1 and IGF2 as well as their receptors in differentiating stem cells. Interestingly, the expression of IGF1 was extremely high in undifferentiated cells and then dropped and gradually rose with endothelial differentiation, while IGF2 expression increased temporally with endothelial differentiation. (**Figure 1**) The expression of IGFR1 and IGFR2 paralleled that of IGF1 and IGF2 ligands. These results are consistent with studies in the literature that have implicated the signaling pathway in promoting stem cell pluripotency as well as differentiation. Based on the results that IGF1 and IGF2 have distinct expression patterns during endothelial differentiation, we investigated their role in vasculogenesis.

**Figure 2:**
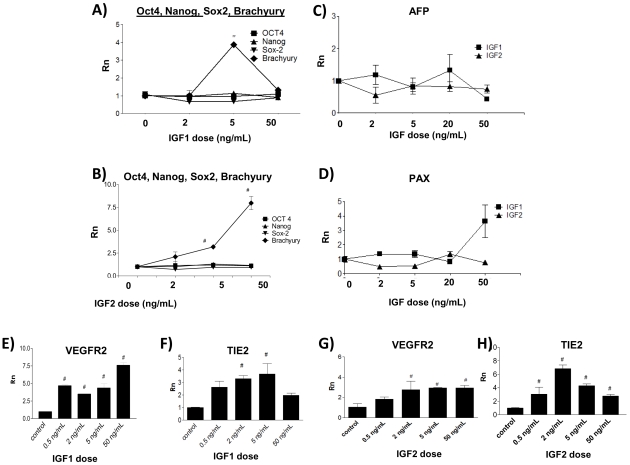
IGF1 and IGF2 promote mesoderm and endothelial differentiation. A–B) Day 3 EB treated with IGF1 and IGF2 display increased mRNA levels of Brachyury and no significant change in Oct4, Nanog, and Sox2. C–D) Levels of AFP and PAX6 are not affected. # denotes P<0.05, Rn denotes normalized reporter. Day 7 EB treated with IGF1 (E,F) and IGF2 (G,H) possess increased levels of endothelial specific markers.

To ascertain the effects of insulin-like growth factors on vasculogenesis, we treated differentiating EB with increasing concentrations of IGF-1 and IGF-2. After 3 hours, media containing IGFs was removed and replaced with fresh media, as continuous exposure to the growth factor caused receptor down-regulation. (not shown) Treatment with IGF-1 or IGF-2 signficantly increased the differentiation of ES into mesoderm compared to control, as measured mRNA levels of mesoderm-specific marker, Brachyury, by quantitative PCR at day 3. The mRNA levels for pluripotency markers, OCT4, Nanog, and Sox2 were not significantly affected by IGF treatment. (**Figure 2A–2B**) Because insulin-like growth factors are known survival factors, we wanted to ensure the effect was mesoderm specific. IGFs did not significantly up-regulate endoderm and ectoderm specific markers, APF and Pax6, leading us to conclude that the proliferating effects of IGF-1 and IGF-2 were mesoderm specific. (**Figure 2C–2D**) The increase in mesoderm generation peaked at approximately 5 ng/mL for IGF-1 but increased with concentrations up to 50 ng/mL for IGF-2. The biphasic concentration response seen with IGF is consistent with similar observations in the case of other angiogenic agents.

### IGF1 and IGF2 treatment Increases Endothelial Differentiation

To look at whether IGF treatment increases differentiation of ES into mature endothelial cells, we considered the mRNA levels of endothelial progenitor specific markers, VEGFR2 and TIE2 in day 7 EB. EB treated with IGF1 or IGF2 expressed significantly higher levels of endothelial markers compared to untreated control. (**Figures 2E–2J**) The optimal concentration of IGF1 or IGF2 to enhance vasculogenesis was approximately 2–5 ng/mL, which resulted in increased expression of endothelial markers as high as 7-fold compared to control. For future experiments, we used 5 ng/mL as the optimal dose of IGFs to induce endothelial differentiation. A marker of functional endothelium, Von Willebrand Factor (VWF) was also specifically upregulated along with markers associated with endothelial progenitor cells, suggesting that IGFs promote development of a mature, functional endothelial cells. (**Figure S1A**) Because IGF promoted differentiation appears to be specific for the mesoderm lineage, we also looked at the mRNA expression of a panel of mesoderm markers at 7 days following daily IGF1 and IGF2 treatment in differentiating EB including those for cardiomyocyte, skeletal myocyte and hematopoietic lineages. Although IGF1 treatment did result in a significant increase in alpha-myosin heavy chain αMHC (muscle specific) and β-globin (erythroid specific) mRNA expression, there was no significant change or a significant decrease in markers of other mesoderm lineages. (**Figure S1B–S1F**)

### Inhibition of IGFR-1 blocks effects of IGF1 and IGF2 treatment and suppresses endothelial differentiation

**Figure 3:**
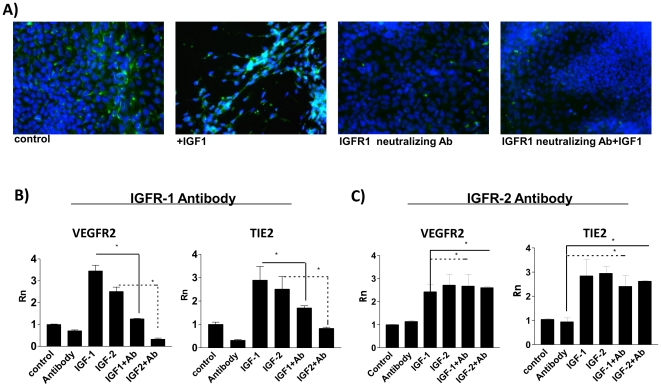
Inhibition of IGFR1, but not IGFR2, inhibits vasculogenesis in differentiating embryoid bodies. A) Tie-2 expression in is decreased in EB treated with IGFR1 neutralizing antibody (nAb IGFR1). Tie-2 expression (FITC), DAPI nuclear counterstain. B) IGFR1 neutralizing antibody treatment (Antibody/Ab) significantly reduces levels of endothelial markers. C) IGFR2 neutralizing antibody treatment had no significant effects on levels of VEGFR2 or Tie-2. * denotes P<0.05 to control, Rn denotes normalized reporter.

To look closer at IGF signaling, we investigated the effects of blocking Insulin-like Growth Factor Receptor 1 (IGFR-1), the major receptor through which IGF-1 and IGF-2 signal. A neutralizing antibody for IGFR-1 significantly inhibited the effects of IGF1 and IGF2 on differentiating EB. Levels of endothelial specific markers are substantially reduced when EB are treated with IGFR-1 antibody, as seen in EB immuno-stained for progenitor and mature endothelial marker TIE2. Differentiated 7 day EB treated with 5 ng/mL IGF1 and IGFR-1 neutralizing antibody exhibited minimal TIE2 staining compared to control cultures wherein regions of TIE2 staining appear within vascular structure outgrowths. (**Figure 3A**). This was confirmed by quantitative PCR, where IGFR-1 neutralizing antibody significantly decreased the endothelial enhancing effects of IGF1 and IGF2, as measured by levels of VEGFR2 and TIE2. (**Figure 3B**) In contrast, IGFR-2 neutralizing antibody failed to exert any significant reduction in IGF-induced vascular differentiation. (**Figure 3C**) These results indicate that the effects of IGFs on mesoderm development and vasculogenesis are mediated specifically through IGFR-1 signaling.

**Figure 4:**
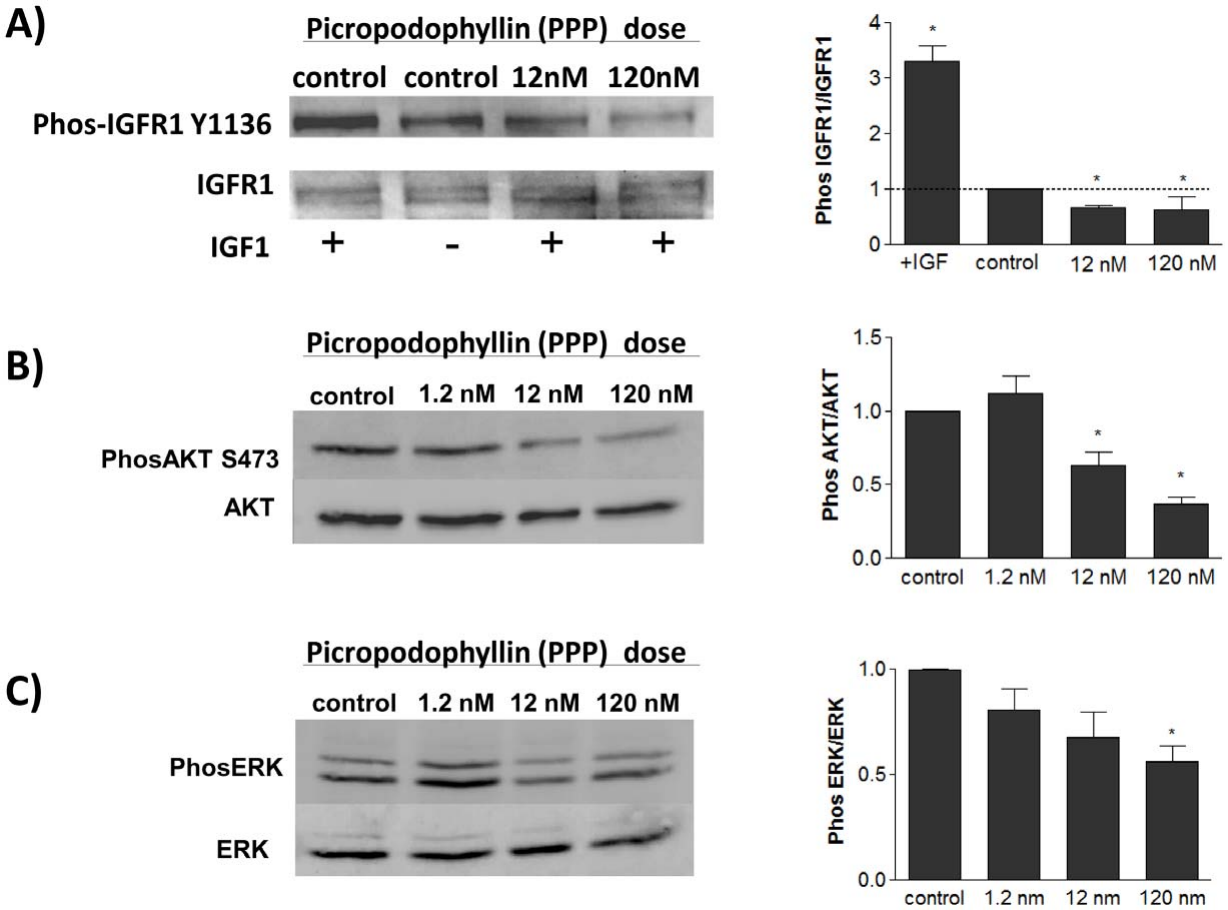
Picropodophyllin treatment decreases IGFR1 signaling and downstream AKT and ERK signaling. A) IGFR1 Y1136 Phosphorylation was inhibited with picropodophyllin (PPP). A random pharmacologic control drug, PHA, had no effect on IGFR1 phosphorylation. B) PPP treatment significantly decreases downstream AKT S473 phosphorylation and ERK p44/p42 phosphorylation (C). * denotes P<0.05 compared to control. (n = 3).

**Figure 5:**
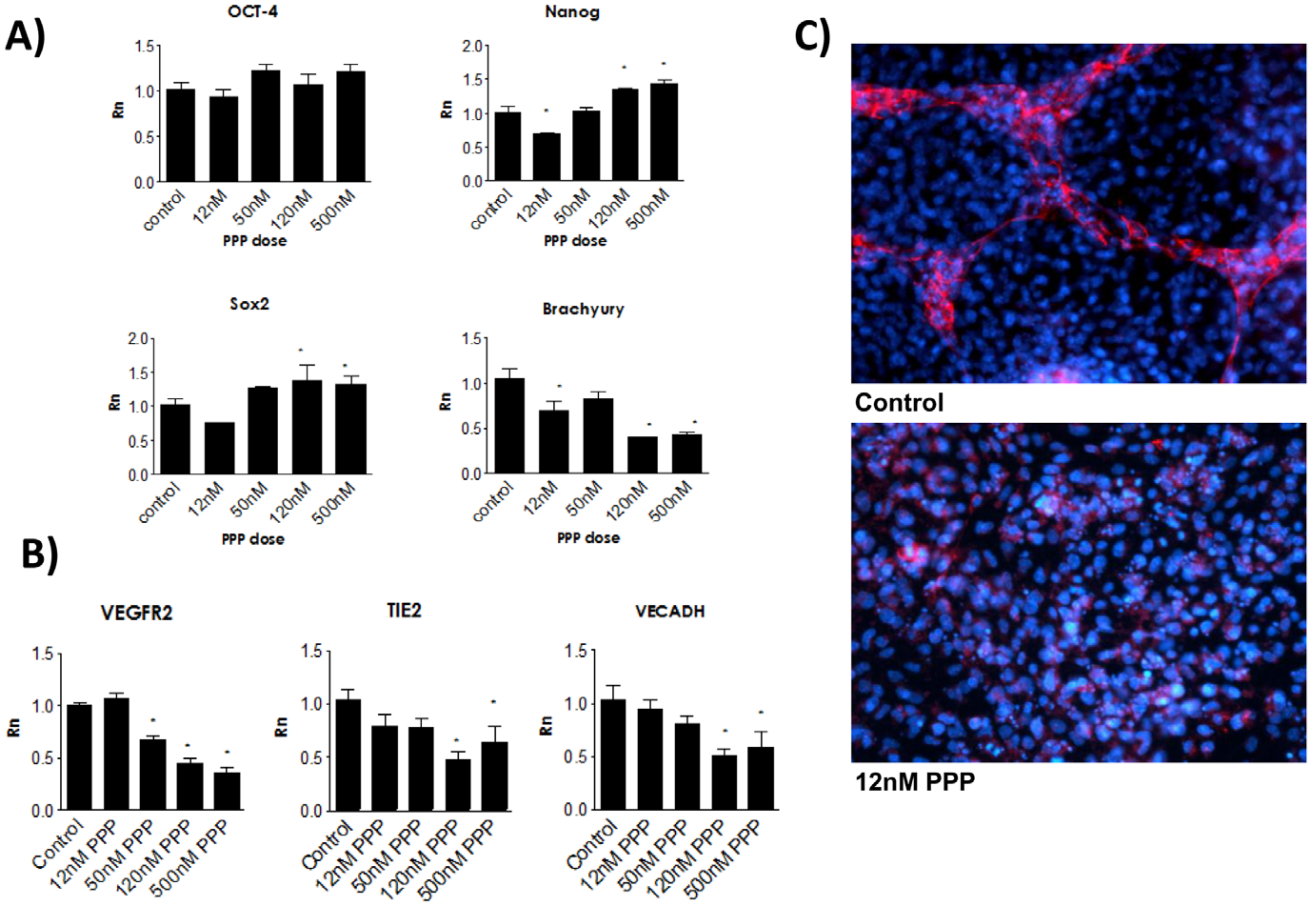
Picropodophyllin treatment inhibits mesoderm and endothelial formation in differentiating embryoid bodies. Day 3 EB treated with PPP have unchanged or increased levels of pluripotency markers significantly decreased levels of Brachyury. B) PPP treatment decreases expression of vascular markers at day 7. C) Differentiated EB cultures treated with PPP and stained for CD31 (TRITC) had strikingly less vessels compared to control on day 10. *denotes P<0.05, Rn denotes normalized reporter.

Picropodophyllin (PPP), is a selective inhibitor of IGFR-1 which binds to its Tyrosine 1136 residue and blocks its capacity for autophosphorylation and downstream signaling. We demonstrated that PPP inhibits phosphorylation of IGFR-1 at the expected phosphorylation site as well as inhibits phosphorylation of downstream signaling targets, AKT and ERK. (**Figure 4**) Treatment of EB with PPP inhibited ES mesoderm differentiation as measured at day 3. Levels of pluripotency markers Nanog and Sox2 were significantly increased, whereas mesoderm marker Brachyury was decreased. (**Figure 5A**) Futhermore, at one week, PPP significantly inhibited endothelial differentiation, as indicated by levels of VEGFR2, TEK, and VE-CADH. (**Figure 5B**) Immunostaining for CD31 additionally revealed that treatment with 12 nM PPP severely decreased the development of vasculature in differentiated embryoid bodies compared to controls at day 10. (**Figure 5C**)

### IGFs promote EB differentiation at various stages in the vasculogenesis pathway

**Figure 6:**
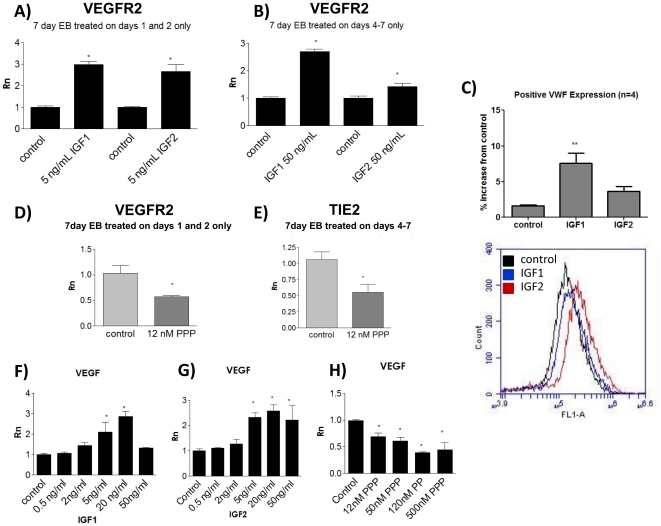
IGFs induce endothelial differentiation at early and late stages of vasculogenesis and increase expression of VEGF. A–B) VEGFR2 and Tie-2 levels at day 7 are significantly increased in EB treated on days 1 and 2 only or on days 4–7 only. C) Treatment with IGF1 or IGF2 post-mesoderm development results in an increase in the percentage of VWF-positive cells compared to control differentiation EB. D–E) PPP treatment on days 1 and 2 only or on days 4–7 only reduced levels of endothelial markers at day 7. Levels of VEGF increased with IGF1 and IGF2 treatment, and decreased with PPP treatment (F–H), at day 3. * denotes P<0.05 compared to control ** P<0.01, Rn denotes normalized reporter.

Based on the above results, we further investigated whether IGFs increase endothelial development by simply increasing mesoderm differentiation, or play at role at other stages of vascular development. Indeed, EB treated with IGFs at early stages of differentiation before mesoderm development, treated on days 1 and 2 only, displayed increased numbers of endothelial cells after one week as measured quantitatively by levels of VEGFR2 mRNA. However, EB treated post-mesoderm development with IGF-1 and IGF-2 also contained increased levels of endothelial cells compared to controls but a higher dose of IGF was required to see this effect. (**Figure 6A–6B**) These results indicate that one of the major means by which IGFs enhance vasculogenesis is by stimulating mesoderm development but that IGFs also enhance endothelial development at later stages in differentiation. To further confirm the IGF-promoted increase in endothelial specific differentiation, we used flow cytometry to quantify the percentage of differentiation endothelial cells when cells were treated with IGFs post-mesoderm development. As measured by positive staining for VWF compared to isotype control, the mean percentage increase of endothelial cells compared to control was 7.56 with IGF1 treatment and 3.67 with IGF2 treatment (n = 4). (**Figure 6C**) Treatment with PPP before day 3 also severely decreased the number of endothelial cells at one week, as did treatment after mesoderm development. (**Figure 6D–6H**) Taken together, the data indicates that IGFs enhance both differentiation of mesoderm, as well as other stages of endothelial lineage differentiation.

### IGFs increase HIF1α stabilization and expression of Vascular Endothelial Growth Factor

**Figure 7:**
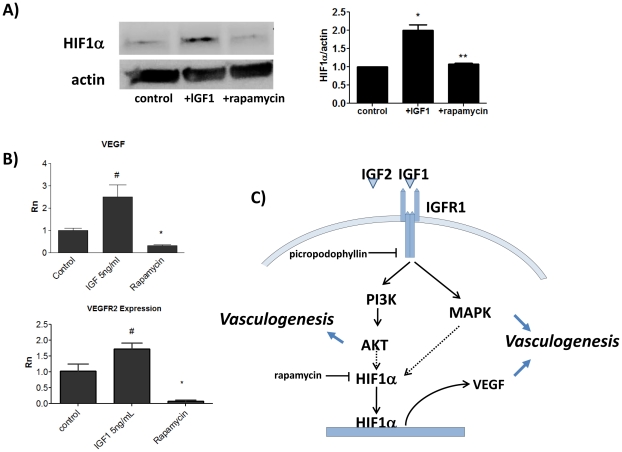
IGF1 increases HIF1α protein levels in differentiating embryoid bodies and inhibition of HIF1α with rapamycin decreases VEGF and VEGFR2 expression in embryoid bodies treated with IGF1. A) HIF1αlevels increase significantly following treatment with IGF1. Rapamycin reduces protein levels when added to cell cultures prior to IGF1 stimulation. * denotes P<0.05 compared to conrol, ** denotes P<0.05 compared to +IGF B) Day 3 EB treated with IGF1 express significantly higher levels of VEGF and VEGFR2 compared with control (# denotes P<0.05 compared with control), which are significantly inhibited by treatment with HIF1α inhibitor rapamycin. (* denotes P<0.05 compared to IGF1 treatment). C) Proposed mechanism of IGF1 and IGF2 stimulated endothelial differentiation.

To further explore the mechanisms underlying IGF-induced vasculogenesis, we studied the effect of IGF treatment on the expression of HIF1α in EB. Previous studies have indicated that IGF can stabilize HIF1α, an important regulator of VEGF expression in neovascularization. [30] As seen in **Figure 7A**, we demonstrated that HIF1α protein levels were significantly increased by approximately two-fold following stimulation with IGF1. Stabilization and translocation of HIF1α to the nucleus is one of the major stimuli for VEGF transcription. [31] Indeed, we confirmed that VEGF expression was significantly increased at day 3 and day 7 following treatment with IGF1 or IGF2, and treatment of EB with PPP decreased VEGF levels (**Figure 6E–6G**). Furthermore, treatment of differentiating cells with rapamycin significantly decreased IGF-induced increase in HIF1α protein levels and inhibited VEGF transcription as well as VEGFR2 levels. (**Figure 7B**) Taken together, our results support a mechanism wherein IGF treatment activates downstream PI3 Kinase and MAP Kinase pathways leading to stabilization of intracellular HIF1α, which in turn up-regulates transcription of VEGF, the critical endothelial cell differentiation factor. (**Figure 7C**)

## Discussion

We have determined, for the first time, the direct effects of IGF1 and IGF2 on vasculogenesis in an embryoid body system (EB). Our results demonstrate that treatment with IGF1 and IGF2 increase the generation of endothelial cells in a spontaneously differentiating EB model. Daily pulse treatment of EB with IGFs caused a significant increase in mesoderm generation which translated to increased differentiation into endothelium compared to control. Interestingly, we did not see a significant increase in mRNA levels for Pax6 or AFP, markers of ectoderm and endoderm, suggesting that the effect is specific for the mesoderm lineage and importantly, and is not simply due to the proliferation of all cell types. To see if this was the reason for the increased number of generated endothelial cells, we treated EB with IGFs only after mesoderm formation. Treatment with IGFs after day 3 also caused a significant increase in endothelial differentiation at one week. These results suggest that insulin-like growth factors play a role in promoting vasculogenesis at both of these critical stages. Although the percentage of differentiated endothelial cells increased by only 7.6% and 3.7% with IGF1 and IGF2 post-mesoderm development, this represents a substantial increase in differentiation considering the most potent vasculogenic factor VEGF was shown to increase differentiation in EB by approximately 10% and further confirms that IGF treatment plays a specific role in the endothelial pathway. [4] Furthermore, although treatment with IGF1 did result in significant increase of some other mesoderm lineage markers, the optimal IGF1 and IGF2 treatment dose for promoting vasculogenesis did not result in increased differentiation of all mesoderm lineages. This corroborates previous studies which have shown that IGFs promote both mesoderm formation [27], and endothelial progenitor cell mobilization and differentiation in functional endothelium. [32] Insulin-like growth factors have previously been shown to promote differentiation of several cell types, including neurons, myocytes and osteoblasts. [33]–[35] IGF in combination with other growth factors like VEGF and FGF, has been shown to induce optimal endothelial differentiation from stem cells, but to our knowledge ours is the first mechanistic study to look directly at IGF-1 and IGF-2 treatment alone using an embryonic stem cell vasculogenesis model. [36]–[37]

Bendall et al. discovered that IGF2 was sufficient in maintaining human ES self-renewal and pluripotency and postulated that a subset of ES generate a source of the ligand to maintain undifferentiated cultures. [28] Chen and Khillan demonstrated that retinol signaling through IGFR1 led to maintenance of self-renewal and pluripotency in mouse ES. [38] Both studies proposed that the downstream PI3K/AKT pathway was critical for these effects. Indeed, stem cells balance a large number of signaling pathways and subtle shifts in signaling can effectively cause a cell to remain pluripotent or progress along a particular differentiation pathway. Proliferation and differentiation of myoblasts upon IGF treatment were shown to utilize separate downstream pathways. The MAPK pathway was critical for optimal proliferation after IGF-1 stimulation, while PI3K signaling was required for differentiation. [33]

The treatment specifics and context play a large role in determining the downstream effects of IGF treatment on ES. For example, we saw a large difference in mRNA levels when cells were treated continuously with IGF or picropodophyllin compared to ‘pulse’ treatment where media was replaced with fresh control media after specified times; continuous treatments caused a significant down-regulation of IGFR1 and IGFR2. Differential expression of IGF binding proteins could also influence the effects of IGF at various cellular stages. Additionally, in our studies, treatment with IGFs or PPP began after 24 hours of culturing cells in low-adherent conditions, when they have likely gained some differentiation capacity. These results indicate that the availability of insulin like growth factors is tightly regulated to control their cellular affects.

We confirmed that one of the mechanisms by which IGF promotes vasculogenesis is by HIF1α-mediated up-regulation of VEGF. Indeed, we demonstrate significant up-regulation of VEGF mRNA after treatment with IGF1 or IGF2. The doses that produced optimal VEGF upregulation corresponded with those that stimulated the most endothelial differentiation. Picropodophyllin, the specific inhibitor of IGFR1, significantly decreased VEGF mRNA levels. VEGF signaling is the decisive factor for mesoderm cells to commit to the endothelial lineage differentiation pathway; it is also a major stimulus for differentiation of endothelial progenitor cells into mature endothelium. Picropodophyllin (PPP) is a cyclolignan that inhibits IGFR1 activation by sterically hindering phosphorylation of adjacent 1135 and 1136 tyrosines on the intracellular receptor domain, while having no effect on the phosphorylation of closely related insulin receptor or non-related tyrosine kinase receptors including FGFR, PDGFR, and EGFR, nor did it down-regulate insulin, VEGFR, EGFR, or PDGFR. [39]–[40] PPP was shown to inhibit IGFR1 signaling in a multiple melanoma mouse model leading to reduced levels of VEGF and a significant reduction in angiogenesis and increased survival. [41] IGF induced up-regulation of VEGF secretion was also blocked by PPP in choroid tumors and Kaposi sarcoma. [42]–[43] IGF1 has been shown to upregulate VEGF in many cell types, including colon cancer, pancreatic cancer, fibroblasts, and osteoblasts. [44]–[45]

HIF1α is a major transcriptional activator of VEGF and VEGF receptors and a major stimulus for embryonic vasculogenesis and neovascularization in embryoid bodies. [46] In normoxic conditions, the protein is degraded by the ubiquitin pathway, but is stabilized in hypoxic conditions and goes on to transcribe genes critical for vasculogenesis, metabolism and cell survival. Inhibition of HIF1α in ES dramatically inhibits levels of VEGF and results in reduced vascular development. [47]–[48] HIF1α synthesis is activated by various oncogenes, cytokines, and growth factors. [31] IGF-1 has been established as a posttranscriptional stimulator of HIF1α accumulation, nuclear translocation, and activity. [30] IGF1 increases HIF1α protein levels and VEGF transcription in Kaposi Sarcoma which is inhibited by treatment with PPP. [49] IGFR1 signaling mediated through downstream AKT and MAPK pathways leads to increased levels of HIF1α and VEGF in pancreatic, colon, and other cancers and therefore was investigated as an anti-angiogenic target. [45] We have shown that IGF1 stimulation of embyroid bodies causes and increase in levels of HIF1α protein in embryoid bodies. Treatment with HIF1α inhibitor rapamycin inhibits intracellular protein levels of HIF1α and blocks IGF-induced increase in VEGF transcription and endothelial progenitor differentiation. This suggests that the HIF1α/VEGF axis is one of the key mechanisms through which IGF1 and IGF2 increase endothelial differentiation. Further, HIF1α has been shown to increase transcription levels of IGF2, resulting in an autocrine IGFR1 activation/VEGF expression loop. [50]

Our study establishes a convincing role for both IGF1 and IGF2 in stimulating a substantial increase in vasculogenesis in embryonic stem cells. Understanding the mechanisms which regulate vascular development in various models, like the EB, can lead to a better understanding of neovascularization *in vivo*. Treatments at various stages of differentiation revealed that IGFR1 signaling promotes differentiation of stem cells into mesoderm, as well as endothelial progenitor cells into mature endothelial cells, indicating that IGFs can emerge as a powerful tool for enabling vascular regeneration therapies.

## Supporting Information

Figure S1 **IGF treatment has different effects on various mesoderm lineage differentiation.** Daily treatment with IGF1 and IGF2 had mixed results on mRNA levels of transcripts specific for various mesoderm lineages: A) Functional endothelial marker, VWF B) Cardiomyocyte transcription factor Nkx2.5 C,D) Muscle specific a-MHC and myogenin E,F) hematopoietic lineage transcripts b-globin and GATA1. (* denotes P<0.05 compared to control)
(TIF)
