# Supplementary figures and images for: Insulin-Like Growth Factors Promote Vasculogenesis in Embryonic Stem Cells
Source: PLoS One. 2012 Feb 21;7(2):e32191. doi: 10.1371/journal.pone.0032191 (PMC3283730; doi:10.1371/journal.pone.0032191)

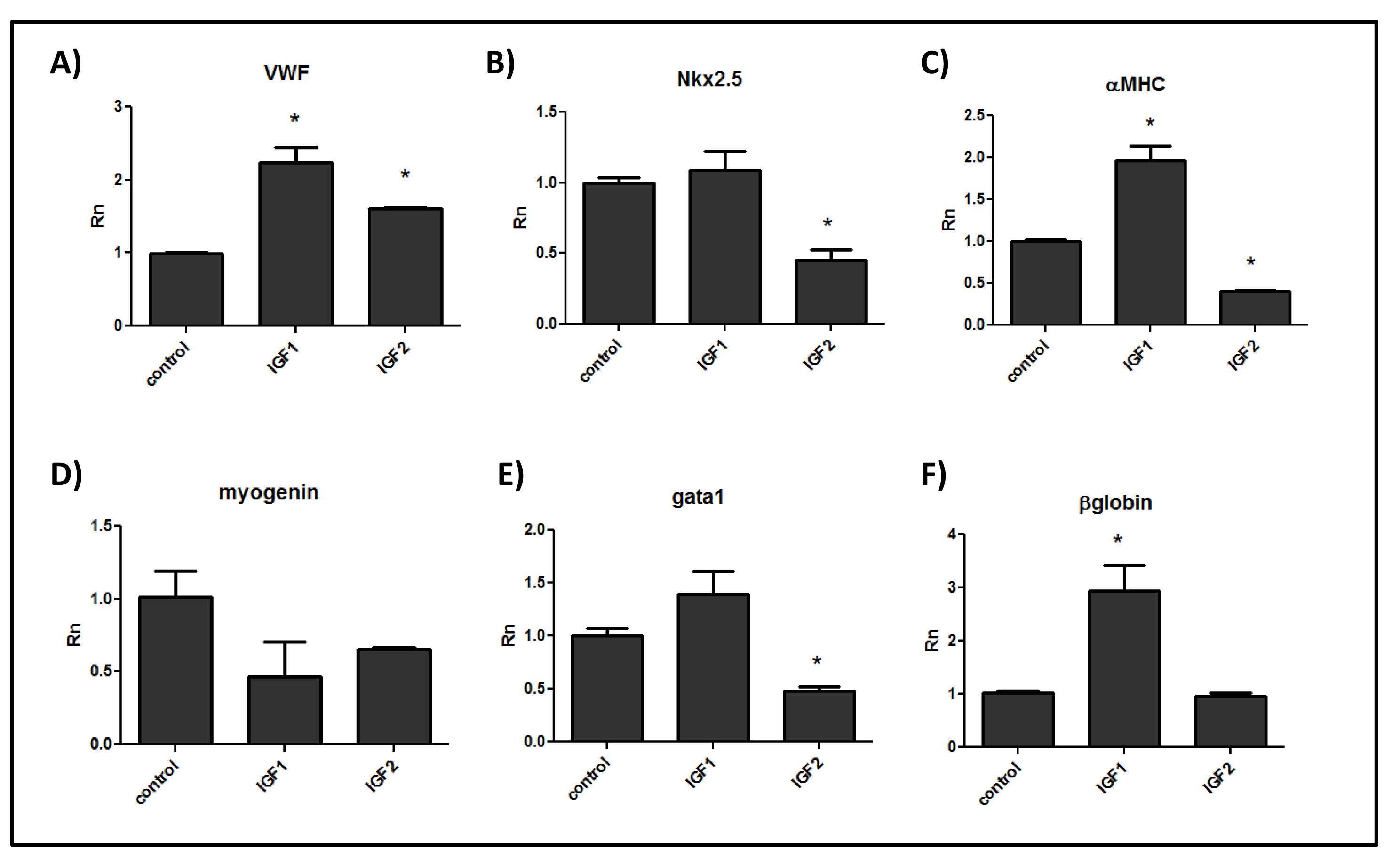

Supplement: Figure S1 — IGF treatment has different effects on various mesoderm lineage differentiation. Daily treatment with IGF1 and IGF2 had mixed results on mRNA levels of transcripts specific for various mesoderm lineages: A) Functional endothelial marker, VWF B) Cardiomyocyte transcription factor Nkx2.5 C,D) Muscle specific a-MHC and myogenin E,F) hematopoietic lineage transcripts b-globin and GATA1. (* denotes P<0.05 compared to control) (TIF) [file pone.0032191.s001.tif]
